# Factors Associated with Neurologists' Provision of MS Patient Care

**DOI:** 10.1155/2014/624790

**Published:** 2014-04-24

**Authors:** Michael T. Halpern, Stephanie M. Teixeira-Poit, Heather Kane, Corey Frost, Michael Keating, Murrey Olmsted

**Affiliations:** ^1^RTI International, 701 13th Street NW No. 750, Washington, DC 20005, USA; ^2^RTI International, 3040 East Cornwallis Road,Research Triangle Park, NC 27709-2194, USA

## Abstract

Neurologists are central to providing quality care for individuals with MS. However, neurologist shortages may restrict access to care for MS patients. To examine factors influencing neurologists' provision of MS care, we surveyed 1,700 US neurologists to assess demographic/practice characteristics, training, and attitudes toward MS care. The study population consisted of 573 respondents: 87 (15.2%) MS subspecialists and 486 (84.8%) “other neurologists,” including subspecialists in other neurology areas (i.e., non-MS) and general neurologists. MS subspecialists indicating they “enjoy interacting with MS patients” had a significantly greater rate of MS patients seen per week. In separate analyses of the “other neurologists” group, the rate of MS patients seen was lower among neurologists in university-based groups or those practicing in major cities; female neurologists; and neurologists who indicated lack of sufficient knowledge regarding MS patient care. Rates of MS patients seen were significantly greater for other neurologists who agreed that MS care involved “ability to improve patient outcomes and quality of life”; “dynamic area with evolving treatment options”; and “enjoy interacting with MS patients.” Understanding factors influencing MS patient care by neurologists and developing policies for appropriate access to care is critical for optimal outcomes among this population.

## 1. Introduction

Neurologists are central to the provision of quality, patient-centered care for individuals with MS. The substantial majority of MS patients depend on neurologists for high quality treatment and education about their condition [1, 2]; for example, MS patients receiving care from neurologists are more likely to receive and use disease-modifying therapies (DMT), participate in rehabilitation clinics, and receive care from rehabilitation specialists (e.g., occupational and physical therapists) and urologists compared with those MS patients receiving care from primary care providers [3]. Neurologists are also critical to quality MS care because of their detailed knowledge of treatment options for MS patients. MS treatment options have become increasingly complicated as the available DMTs increase and their benefits and safety profiles remain unclear [1, 4].

Among neurologists, MS subspecialists have the greatest familiarity with MS patient care. Little information is available regarding difference in treatment patterns or patient perceptions of care among individuals with MS receiving care from MS subspecialists versus from other neurologists. Although MS patients receiving care from neurologists and from MS subspecialists have been reported to experience similar use of healthcare services, those receiving care from MS subspecialists reported greater knowledge about and current use of a particular DMTs (i.e., interferon beta-1b), expressed more confidence in their physicians, and indicated greater participation in nondrug research [5].

Although neurologists are critical to providing MS care, shortages among the neurologist workforce (including both MS subspecialists and other neurologists) may restrict access to neurologists among individuals with MS [6]. Anecdotal evidence from patient groups suggests that MS patients experience difficulty in gaining access to appropriate care, especially from MS subspecialists; new patients may experience substantial waits and difficulties in finding available neurologists. Understanding the factors that influence the number of MS patients seen by neurologists can aid in identifying challenges that MS subspecialists and other neurologists encounter in seeing MS patients and can suggest potential solutions for resolving these challenges. This study examines results from a recently completed study assessing factors associated with the number of MS patients seen by MS subspecialists and other neurologists.

## 2. Materials and Methods

### 2.1. Instrument Development

We developed the MS Physician Workforce Neurologist Survey initially by reviewing previously developed physician career surveys to identify items relevant to assessing neurologists' care for MS patients. We selected and adapted existing questions and crafted new items to create a draft survey, which included questions on respondents' demographic characteristics, medical training, attitudes toward providing MS patient care, MS subspecialist status, practice characteristics, and numbers of MS patients seen.

To ensure comprehensiveness and usability of the survey, we sought input from an advisory panel of experts identified for this study and the American Academy of Neurology (AAN) MS Section Executive Committee. The advisory panel, including neurologists, reviewed and provided comments on the survey, which was then revised based on their feedback. Members of the AAN MS Section Executive Committee then reviewed the survey to ensure that it was feasible for AAN members to complete. This final version of survey was approved by the RTI International Institutional Review Board prior to distribution. The survey is available from the authors on request.

### 2.2. Study Population and Survey Administration

The sampling frame consisted of neurologists practicing in the United States who are members of the AAN. We collaborated with the AAN, which administered the survey and collected responses. The AAN contacted a random sample of 1,700 neurologists who resided in the United States and were members of the AAN, excluding retired members; those in medical school, residency, or fellowship; and AAN committee members who participated in survey review. Seven respondents were later removed from the sample because they were not in practice or did not see adult patients.

The AAN administered the survey via email as well as postal mail or fax to the sample of neurologists in January 2012. The AAN sent neurologists an email describing the study and including a hyperlink to the online version of the survey. The paper version also included a cover letter signed by the Chair of the AAN MS Section. AAN staff tracked responses to determine whether invited neurologists submitted surveys and, for nonresponders, distributed two email reminders to participate. Of the 1,693 neurologists invited to participate, 662 submitted responses (response rate of 39.1%).

### 2.3. Study Data

We used identification numbers to merge (deidentified) neurologists' survey responses with demographic information and practice characteristics from the* 2008 AAN Member Census*. From the* Census*, practice arrangement were categorized as solo practice, neurology group, multispecialty group, university based group, other, and unknown. The “other” category included staff-model HMO; government hospital or clinic; and other public or private hospital or clinic setting.

### 2.4. Study Variables

The dependent variable for this analysis was self-reported total number of MS patients seen in an average week. Independent variables included demographic characteristics, practice characteristics, and attitudes toward MS patient care. Self-reported demographic characteristics included age group (categorized in quartiles), sex, race (White, Asian, and other race including Black/African American, American Indian/Alaska Native, and Native Hawaiian/other Pacific Islander groups combined because of small numbers), ethnicity (Hispanic/Latino or not), and year began medical practice (categorized in quartiles). Urban/rural designation of practice area was coded into three categories: within a major city, suburban or moderate-sized city, and rural area or small city.

Survey respondents were categorized as MS subspecialists using two sets of criteria. First, based on fellowship training those who had completed a MS fellowship and reported seeing, on average, at least one MS patient per week were classified as MS subspecialists. As neurologists may also subspecialize in MS patient care without completing a MS fellowship, respondents who reported seeing, on average, more than 12 MS patients per week (the median number of MS patient seen per week among neurologists who did not complete a MS fellowship but considered themselves to be MS subspecialists) were also classified as MS subspecialists.

The survey captured information on 17 attitudes toward MS patient care. Ten of these attitudes reflected factors that could limit the number of MS patients seen by respondents, while seven corresponded to positive aspects of providing MS care (Table 2). Attitudes were coded dichotomously (i.e., zero or one), based on whether a respondent indicated that each attitude was applicable to his or her practice.

### 2.5. Analyses

We computed bivariate analyses to examine factors associated with number of MS patients seen. This dependent variable (number of MS patients seen) was examined separately for MS subspecialists versus other neurologists (i.e., general neurologists and subspecialists in areas other than MS, referred to as “non-MS subspecialists”). Since this dependent variable was not normally distributed (Shapiro-Wilk *W* tests for normality, *P* < 0.001), we computed nonparametric bivariate analyses to examine factors associated with MS patient seen using the Wilcoxon-Mann-Whitney tests for independent variables with two categories and the Kruskal-Wallis tests for independent variables with three or more categories.

We also conducted bivariate analyses to compare characteristics and attitudes of other neurologists (non-MS subspecialists and general neurologists) who did not see any MS patients with those who saw MS patients, using *χ* ^2^ tests or, because of small cell sizes, Fisher's exact tests to examine relationships between discrete variables (e.g., sex) and the proportion seeing any MS patients.

We performed multivariate regression analyses to examine the association of neurologist characteristics and attitudes with numbers of MS patients seen while controlling for other potentially associated factors. Since residuals for the dependent variables were not normally distributed, we used negative binomial regression models with robust standard errors to identify predictors of number of MS patients seen. As factors associated with numbers of MS patients seen may differ between MS subspecialists and other neurologists, regression analyses were performed separately for these two populations. Results from the negative binomial regressions are presented as incidence rate ratios (IRRs), that is, the “rate” of seeing individuals with MS associated with a predictor variable relative to the reference case for that variable. IRRs less than 1.0 indicate decreased rates of seeing MS patients compared with the reference case, while IRRs greater than 1.0 indicate increased rates. Regression models included all independent variables (i.e., survey respondents' characteristics) that were significantly (*P* < 0.05) or marginally significantly (0.05 < *P* < 0.10) associated with the number of MS patients seen in bivariate analyses (Tables 1 and 2). All variables included in regression analyses were examined for multicollinearity (VIF > 5) prior to inclusion in the final model. Because of multicollinearity with age, the variable “year began medical practice” was removed from the regression model predicting MS patients seen per week among general neurologists and non-MS subspecialists (as age was not significantly associated with number of MS patients seen among MS subspecialists, age was not included in that regression model). Missing data generally comprised less than 3% of responses; individuals with missing data for descriptive, bivariate, or regression analyses were excluded from those analyses.

## 3. Results

### 3.1. Population Characteristics and MS Care Attitudes

After excluding neurologists who did not provide information about their subspecialty status, the study population consisted of 573 neurologists. Of these neurologists, 87 (15.2%) were categorized as MS subspecialists and 486 (84.8%) were “other neurologists,” including subspecialists in other areas of neurology (i.e., not MS) and general neurologists (Table 1). Among the 87 MS subspecialists, 26 (approximately 30%) were classified as MS subspecialists based on having completed an MS fellowship, while the remaining 61 were classified in this group based on seeing, on average, more than 12 MS patients per week.

After excluding neurologists who did not respond to the questions for other specified dependent variables, MS subspecialists saw approximately 25 MS patients per week on average (median = 20). Other neurologists saw approximately three MS patients per week on average (median = 3). MS subspecialists and other neurologists indicated substantial differences in attitudes regarding MS patient care (Table 2).

### 3.2. Characteristics Associated with Number of MS Patients Seen per Week

MS subspecialists saw, on average, more MS patients per week if they indicated the following attitudes: “enjoy interacting with MS patients” (*P* = 0.001), “care involving a multidisciplinary team” (marginally significant), and “personal connection to individuals with MS who are not your patients” (marginally significant) (Table 2). MS subspecialists who began medical practice between 1972 and 1985 were also marginally more likely to see more MS patients per week than MS subspecialists who began practice later. Practice arrangement, physician age, sex, race, ethnicity, urban/rural practice area, and the remaining attitudes toward providing MS patient care were not significantly associated with number of MS patients seen (Table 2).

Among survey respondents in the “other neurologist” group, general neurologists saw significantly more MS patients per week than did non-MS subspecialists (4.4 versus 3.2, resp., *P* < 0.005) (Table 2). Physicians in a neurology or multispecialty groups also saw more MS patients that did those in solo practice. Younger neurologists, female neurologists, those practicing for fewer years, and those based within a major city saw fewer MS patients.

General neurologist and non-MS subspecialist respondents who indicated the following attitudes saw significantly fewer MS patients per week: “lack of sufficient knowledge to feel comfortable caring for this patient population”; “lack of sufficient knowledge regarding newer disease-modifying drugs”; and “seldom encounter MS patients” (Table 2). In contrast, neurologists who indicated “insufficient reimbursement for time involved” surprisingly saw more MS patients. Other neurologist respondents saw more MS patients if they agreed that MS patient care involved “ability to improve patient outcomes and quality of life,” “dynamic area with evolving treatment options,” “care involving a multidisciplinary approach,” “enjoy interacting with MS patients,” and “research opportunities” (marginally significant). As with MS subspecialists, general neurologist and non-MS subspecialist respondents based within a major city saw fewer MS patients. Race, ethnicity, and the remaining attitudes toward providing MS patient care were not significantly associated with number of MS patients seen.

### 3.3. Multivariate Regression Analyses of Number of MS Patients Seen

We used negative binomial regressions (as presented in Section 2) to examine the association of neurologist characteristics and attitudes toward MS care with the number of MS patients seen per week while controlling for other potentially associated factors. Analyses were performed separately for MS subspecialists (Table 3) and general neurologists/non-MS subspecialists (Table 4). All survey items that had significant or marginally significant associations with number of MS patients seen in bivariate analyses (Table 2) were included as independent variables in these regression models.

Among MS subspecialists, the only factor significantly associated with number of MS patients seen per week in regression analysis was the attitude “enjoy interacting with MS patients”; MS subspecialists who indicated this attitude had a rate of MS patients seen per week that was 1.6 times greater than did those not indicating this attitude. MS subspecialists who began medical practice between 1986 and 1993 saw fewer MS patients than did those who had been practicing longer, although this difference was only marginally significant (*P* = 0.060).

In regressions analyses of the “other neurologists” group, the rate of MS patients seen per week among neurologists in a university-based group was approximately 60% (0.59 times) the rate for those in a solo practice. The rate of MS patient seen among female other neurologists was approximately 80% the rate among males. The rates of MS patients seen were significantly lower for non-MS subspecialists/general neurologists who indicated “lack of sufficient knowledge to feel comfortable caring for this patient population” and “seldom encounter MS patients.” The rates of MS patients seen were significantly greater for other neurologists who agreed with the following attitudes toward MS patient care: “ability to improve patient outcomes and quality of life,” “dynamic area with evolving treatment options,” and “enjoy interacting with MS patients.” Among other neurologists practicing in rural areas or small cities, the rate of MS patients seen per week was 1.30 times greater than among those practicing within major cities; practicing in suburban areas or moderate-sized cities was marginally associated with seeing more MS patients.

**Figure 1:**
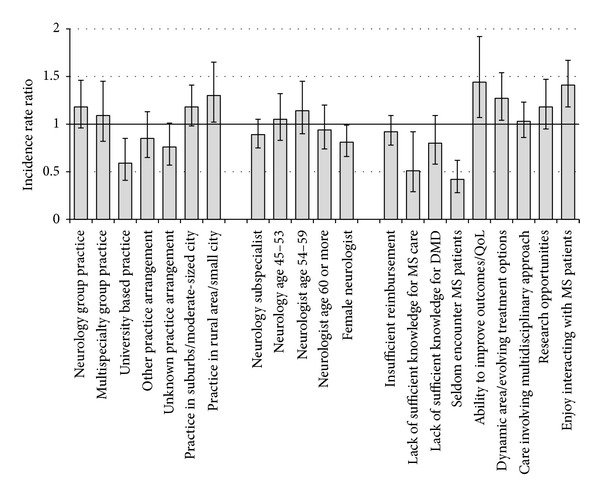
Incidence rate ratios for MS patients seen per week among non-MS subspecialists/general neurologists.

Figure 1 graphically presents results from Table 4, illustrating the incidence rate ratios (IRR) and 95% confidence intervals for the association of non-MS subspecialist/general neurologist characteristics and attitudes with the number of MS patients seen per week. The solid horizontal line indicates IRR of 1.0, corresponding to no significant difference from the reference group for that characteristics or attitude. Results are presented in three groups. Among neurology practice characteristics, those in university-based practices had a significantly decreased rate of seeing MS patients, while those in small city/rural practices had increased rates. Examining physician characteristics, female neurologist had significantly decreased rates of seeing MS patients. Finally, for attitudes towards MS patient care, “lack of sufficient knowledge for MS care” and “seldom encounter MS patients” were associated with decreased rates of MS patients seen while “ability to improve outcomes/quality of life,” “dynamic area with evolving treatment options,” and “enjoy interacting with MS patients” were associated with increased rates.

## 4. Discussion

To our knowledge, this is the first published study examining factors influencing the numbers of MS patients seen among a broad sample of US neurologists. Given the importance of neurologist care for MS [2, 3, 7], barriers to access neurologists could have substantial impacts on symptom control, disease progression, and quality of life among individuals with MS. Several studies have described barriers that individuals with MS may experience in receiving needed care. For example, MS patients in rural areas have been reported to have more limited access to MS subspecialists and related care services and had longer travel times than did patients in urban areas to receive specialized MS care [3, 8, 9]. Racial and ethnic disparities are also barriers to MS patient care: African Americans are less likely to have been treated by an MS subspecialist; Latinos are less likely to have received rehabilitation care compared to Caucasians [10]. These barriers to care may have direct consequences on patient outcomes; for example, individuals with MS residing in rural areas had significantly greater reductions in their physical components of health-related quality of life [11].

We found that MS subspecialists responding to this study's survey saw approximately 25 MS patients per week, while other neurologists saw approximately 3.4 patients per week. It is not surprising that general neurologists and neurologists who subspecialize in clinical areas other than MS see, on average, fewer MS patients than do MS subspecialists. However, as the population of individuals with MS in the US continues to increase and only limited numbers of MS subspecialists (particularly outside of large urban/suburban locations) are available to serve MS patients, more MS patients may seek care from these other neurologists. The results presented in this study, providing information on how much care these other neurologists are currently providing for MS patients, may be useful for estimating the future capacity of the neurologist workforce for care for individuals with MS.

Among MS subspecialists, we identified only one factor that was significantly associated with number of MS patients seen per week in multivariate regressions (Table 3). Those who indicated that they enjoyed interacting with MS patients saw more individuals with MS. This may relate to “burnout” among MS subspecialists; that is, subspecialists who do not enjoy interacting with MS patients likely experience less enjoyment from providing medical care and instead may focus on other activities (e.g., teaching or administration) and see fewer individuals with MS. Shanafelt et al. [12] reported that neurologists had the third highest rate of burnout among all physician specialties.

More factors were found to be significantly associated with numbers of MS patients seen among other neurologists (i.e., general neurologists and non-MS subspecialists) (Table 4). Neurologists in university-based practices had significantly lower rates of seeing MS patients compared with the reference group, neurologists in solo practice. This may reflect the nonpatient care activities that are required of university neurologists (including teaching, research, and administration) and the likelihood that many of the “other neurologists” at universities specialized in care for patients with conditions other than MS.

Several attitudes toward providing MS patient care were also associated with numbers of MS patients seen among the “other neurologists.” Respondents who indicated that they lack sufficient MS knowledge or seldom encounter MS patients saw fewer MS patients. These attitudes may be linked; that is, physicians who feel they lack sufficient knowledge may be less likely to have MS patients referred to them. Among physicians who feel they lack sufficient knowledge regarding MS care, increasing knowledge may increase their interest and willingness to see MS patients. In contrast, those who indicated the ability to improve MS patient outcomes and quality of life, considered MS a dynamic area with evolving treatment options, or indicated that they enjoy interacting with MS patients were more likely to see more MS patients. These attitudes likely reflect better knowledge regarding MS care. For example, given the recent (and expected future) development of new MS disease modifying therapies [1, 4], this is clearly a dynamic area with the ability to improve patient outcomes. Knowledge of these newer treatment options as well as familiarity with the benefits of multidisciplinary approaches to MS care will likely increase physician interest and enjoyment in providing care to MS patients. Educational programs to increase neurologist familiarity with MS care may strengthen these positive attitudes and increase access to care for MS patients. Improving general neurologists' knowledge of new treatment options may also enhance MS patient care, as general neurologists may be less likely to discuss newer treatment options than are MS subspecialists [5].

Surprisingly, other neurologists who practice in small cities or rural areas were marginally significantly more likely to see more MS patients than were those practicing in major cities. This appears to contradict previous research findings that MS patients in rural areas have decreased access to care and are more likely to choose other providers (e.g., primary care providers) to direct their care [3, 8]. However, these findings may not be in conflict. MS patients in rural areas likely have few neurologists available; they may therefore be more likely to see neurologists who practice in their local area rather than going to farther away MS subspecialists. Thus, non-MS subspecialist neurologists outside of large urban areas may be more likely to see MS patients as there are no MS centers or MS subspecialists easily available to provide this care.

There are a number of limitations associated with this study. Although the population of neurologists invited to participate in the survey was randomly selected from the relevant members of the AAN, only 39% responded to the survey; this group may not be representative of U.S. neurologists in general. All information provided by survey participants was by self-report; we did not attend to validate any responses. In addition, as with all surveys, we limited the number of items asked to minimize respondent burden. There are likely additional factors that influence the number of MS patients seen by both MS subspecialists and other neurologists that were not captured in our survey. Finally, as our survey data are cross-sectional (i.e., collected at one time point), we are unable to assess causality in the analysis results. For example, we can only determine that an attitude of enjoy interacting of MS patients is associated with seeing more MS patients; we cannot determine whether this attitude preceded initiation of providing MS patient care or whether the attitude was generated based on providing MS patient care (or both). Future studies could involve longitudinal data collection from a cohort of neurology residents, to examine changes in attitudes and subspecialization decisions over time to assess causal relationships in this physician population.

Despite these limitations, this study provides important information on factors affecting numbers of MS patients seen among the neurologist workforce and identifies potential actions to increase access to care for this population.

## 5. Conclusions

Prior studies have suggested current or impending shortages among neurologists [6]. Among individuals with MS, who may require frequent interactions with neurologists, barriers to access care may affect symptom control, quality of life, and potentially even survival. Our results indicate that several factors are associated with decreased provision of MS patient care among “other neurologists” (i.e., general neurologists and neurologists subspecializing in areas other than MS). These factors include demographic and practice characteristics (female neurologists, those in university-based practices, and those practice in major cities were less likely to provide MS patient care) and attitudes towards MS care. The significant associations of attitudes towards MS care with number of MS patients seen by neurologists suggest that increasing neurologist knowledge about treatments for individuals with MS may decrease barriers to seeing more patients. To ensure optimal health outcomes among individuals with MS, it is critical both to gain better insights regarding factors influencing provision of MS patient care by neurologists and to develop policies that ensure appropriate access to high-quality MS care.

## Supplementary Material

MS Physician Workforce Neurologist Survey
The MS Physician Workforce Neurologist Survey was developed based on previous physician surveys, feedback from this study's Advisory Panel, and comments from members of the American Academy of Neurology (AAN) MS Section Executive Committee. This survey collected information from practicing neurologists on attitudes towards providing care for individuals with MS; attitudes towards neurologists who subspecialize in MS care; interactions with MS patients during residency training; current practice characteristics; and professional life satisfaction.

## Figures and Tables

**Table 1:** Descriptive statistics for study populations.

	MS subspecialists^†^		Non-MS subspecialists and general neurologists	
	Number	%	Number	%
Total study population	87	15.18	486	84.82
Subspecialty status:				
Non-MS subspecialist	—	—	364	75.83
General neurologists	—	—	116	24.17
Practice arrangement:				
Solo practice	16	18.39	123	25.31
Neurology group	29	33.33	121	24.90
Multispecialty group	11	12.64	48	9.88
University based group	17	19.54	61	12.55
Other^‡^	5	5.75	56	11.52
Unknown	9	10.34	77	15.84

Demographic characteristics				
Age:				
44 years or less	16	18.60	124	26.05
45 to 53 years	31	36.05	121	25.42
54 to 59 years	25	29.07	118	24.79
60 years or more	14	16.28	113	23.74
Sex:				
Male	61	70.11	344	71.07
Female	26	29.89	140	28.93
White:				
Yes	67	88.16	305	76.63
No	9	11.84	93	23.37
Asian:				
Yes	9	11.84	83	20.85
No	67	88.16	315	79.15
Other race:				
Yes	0	0.00	13	3.27
No	76	100.00	385	96.73
Hispanic or Latino:				
Yes	4	5.41	24	6.25
No	70	94.59	360	93.75
Year began medical practice after completing medical training:				
1972 to 1985	15	17.24	123	25.84
1986 to 1993	32	36.78	103	21.64
1994 to 2001	27	31.03	126	26.47
2002 to 2011	13	14.94	124	26.05

Limiting attitudes towards providing MS patient care				
Care for MS patients takes too much time:				
Yes	36	41.38	87	17.90
No	51	58.62	399	82.10
Insufficient reimbursement for time involved:				
Yes	39	44.83	116	23.87
No	48	55.17	370	76.13
Lack of sufficient knowledge to feel comfortable caring for this patient population:				
Yes	0	0.00	32	6.58
No	87	100.00	454	93.42
Lack of sufficient knowledge regarding newer disease-modifying drugs:				
Yes	1	1.15	59	12.14
No	86	98.85	427	87.86
Lack of special personnel (nurses, social workers, etc.):				
Yes	24	27.59	115	23.66
No	63	72.41	371	76.34
Little can be done to improve MS patients' outcomes:				
Yes	0	0.00	2	0.41
No	87	100.00	484	99.59
Seldom encounter MS patients:				
Yes	0	0.00	70	14.40
No	87	100.00	416	85.60
MS patients are often difficult to treat:				
Yes	6	6.90	48	9.88
No	81	93.10	438	90.12
MS patients often have multiple comorbidities:				
Yes	20	22.99	70	14.40
No	67	77.01	416	85.60
Providing care to MS patients is not sustainable in my practice environment due to time or reimbursement constraints:				
Yes	8	9.20	49	10.08
No	79	90.80	437	89.92

Positive attitudes towards providing MS patient care				
Ability to improve patient outcomes and quality of life:				
Yes	81	93.10	382	78.60
No	6	6.90	104	21.40
Dynamic area with evolving treatment options:				
Yes	77	88.51	270	55.56
No	10	11.49	216	44.44
Care involving a multidisciplinary approach:				
Yes	52	59.77	116	23.87
No	35	40.23	370	76.13
Research opportunities:				
Yes	54	62.07	60	12.35
No	33	37.93	426	87.65
Personal connection to individuals with MS who are not your patients:				
Yes	16	18.39	33	6.79
No	71	81.61	453	93.21
Enjoy interacting with MS patients:				
Yes	62	71.26	173	35.60
No	25	28.74	313	64.40
Community of dedicated professional colleagues with which to interact:				
Yes	43	49.43	100	20.58
No	44	50.57	386	79.42

Practice characteristics				
Area in which you are practicing:				
Within a major city (population greater than 250,000)	50	58.14	234	48.45
Suburban or moderate-sized city (population 50,000 to 250,000)	29	33.72	181	37.47
Rural area or small city (population less than 50,000)	7	8.14	68	14.08

^†^MS subspecialists answered “Yes, and I chose a fellowship in MS” to question “Did you consider a fellowship in MS as subspecialty training?” and, on average, saw at least one MS patients per week. Respondents who did not complete an MS fellowship were categorized as MS subspecialists if indicated that they saw, on average, more than 12 patients per week.

^‡^The “other” category includes neurologists who responded “staff-model HMO,” “government hospital or clinic,” or “other public or private hospital or clinic setting” to question “Indicate in which practice arrangement you spend the majority of your clinical time.”

**Table 2:** MS patients seen per week for MS subspecialists versus Non-MS subspecialists/general neurologists.

	MS subspecialists^†^			Non-MS subspecialists and general neurologists				
	Mean number of MS patients seen per week	Standard deviation	*P* value for difference in means for number of MS patients seen by IV if MS subspecialist*	Mean number of MS patients seen per week	Standard deviation	*P* value for difference in means for number of MS patients seen by IV if non-MS subspecialist or general neurologist*	% seeing zero MS patients per week	*P* value for difference in %**
Total study population	25.33	16.51	—	3.47	3.30	—	20.99	—
Subspecialty status								
Non-MS subspecialist	—	—	—	3.16	3.17	0.0003	23.90	0.003
General neurologists	—	—	—	4.43	3.42		11.21	
Practice arrangement								
Solo practice	23.19	10.55	0.5788	3.92	3.35	0.0001	12.20	0.000
Neurology group	25.86	19.15		4.59	3.46		11.57	
Multispecialty group	28.82	15.70		4.40	3.60		16.67	
University based group	24.88	18.87		1.52	2.12		54.10	
Other^‡^	15.50	10.21		2.94	2.93		16.07	
Unknown	28.33	16.04		2.28	2.71		29.87	

Demographic characteristics								
Age								
44 years or less	28.27	19.25	0.2289	2.79	3.24	0.0047	29.03	0.026
45 to 53 years	24.74	15.80		3.64	3.23		19.01	
54 to 59 years	22.12	14.58		4.17	3.46		15.25	
60 years or more	30.14	18.55		3.56	3.17		15.93	
Sex								
Male	25.42	17.46	0.9281	3.72	3.33	0.0042	18.60	0.055
Female	25.12	14.39		2.86	3.16		26.43	
White								
Yes	24.67	16.39	0.7982	3.78	3.44	0.3742	20.00	0.714
No	24.44	19.11		3.36	3.22		18.28	
Asian								
Yes	24.44	19.11	0.7982	3.37	3.14	0.4811	18.07	0.694
No	24.67	16.39		3.77	3.45		20.00	
Other race								
Yes	—	—	—	3.50	4.15	0.5701	23.08	0.725
No	24.64	16.59		3.69	3.37		19.48	
Hispanic or Latino								
Yes	27.50	10.41	0.3782	2.61	2.69	0.1598	25.00	0.485
No	25.41	17.19		3.68	3.34		19.17	
Year began medical practice after completing medical training								
1972 to 1985	33.00	18.84	0.0816	3.85	3.49	0.0020	16.26	0.071
1986 to 1993	22.25	13.63		3.51	3.33		22.33	
1994 to 2001	25.73	16.04		3.92	3.26		18.25	
2002 to 2011	23.23	19.89		2.55	2.99		29.03	

Negative attitudes towards providing MS patient care								
Care for MS patients takes too much time								
Yes	26.50	19.34	0.8910	3.64	3.26	0.3922	14.94	0.126
No	24.48	14.27		3.43	3.32		22.31	
Insufficient reimbursement for time involved								
Yes	28.82	19.13	0.1157	4.15	3.33	0.0051	11.21	0.003
No	22.43	13.49		3.26	3.27		24.05	
Lack of sufficient knowledge to feel comfortable caring for this patient population								
Yes	—	—	—	0.90	1.47	0.0000	56.25	0.000
No	25.33	16.51		3.66	3.32		18.50	
Lack of sufficient knowledge regarding newer disease-modifying drugs								
Yes	25.00	—	0.5968	1.76	2.19	0.0000	33.90	0.009
No	25.33	16.60		3.70	3.36		19.20	
Lack of special personnel (nurses, social workers, etc.)								
Yes	25.50	19.47	0.4365	3.23	3.04	0.6404	16.52	0.178
No	25.26	15.38		3.55	3.38		22.37	
Little can be done to improve MS patients' outcomes								
Yes	—	—	—	1.00	—	0.3370	0.00	1.000
No	25.33	16.51		3.48	3.31		21.07	
Seldom encounter MS patients								
Yes	—	—	—	1.06	1.88	0.0000	48.57	0.000
No	25.33	16.51		3.87	3.32		16.35	
MS patients are often difficult to treat								
Yes	32.50	19.94	0.3290	3.04	2.87	0.5750	16.67	0.439
No	24.79	16.24		3.52	3.35		21.46	
MS patients often have multiple comorbidities								
Yes	25.85	14.34	0.6909	3.47	2.82	0.4041	10.00	0.015
No	25.17	17.21		3.47	3.38		22.84	
Providing care to MS patients is not sustainable in my practice environment due to time or reimbursement constraints								
Yes	28.75	13.02	0.1764	3.60	3.30	0.7362	18.37	0.635
No	24.97	16.85		3.46	3.31		21.28	

Positive attitudes towards providing MS patient care								
Ability to improve patient outcomes and quality of life								
Yes	25.13	16.55	0.6074	3.94	3.26	0.0000	12.57	0.000
No	28.00	17.15		1.73	2.88		51.92	
Dynamic area with evolving treatment options								
Yes	25.95	16.76	0.1779	4.40	3.37	0.0000	10.74	0.000
No	20.60	14.32		2.30	2.81		33.80	
Care involving a multidisciplinary approach								
Yes	27.96	18.29	0.0952	4.02	3.43	0.0286	13.79	0.029
No	21.29	12.53		3.30	3.25		23.24	
Research opportunities								
Yes	27.45	18.67	0.1364	4.11	3.23	0.0621	16.67	0.380
No	21.91	11.74		3.38	3.31		21.60	
Personal connection to individuals with MS who are not your patients								
Yes	30.69	17.51	0.0676	4.16	3.22	0.1402	15.15	0.394
No	24.10	16.15		3.42	3.31		21.41	
Enjoy interacting with MS patients								
Yes	28.92	17.98	0.0010	4.79	3.36	0.0000	6.94	0.000
No	16.56	6.64		2.71	3.03		28.75	
Community of dedicated professional colleagues with which to interact								
Yes	25.63	17.73	0.8752	3.59	3.29	0.5766	20.00	0.786
No	25.02	15.39		3.44	3.31		21.24	

Practice characteristics								
Area in which you are practicing								
Within a major city (population greater than 250,000)	27.20	18.36	0.5061	2.92	3.07	0.0017	28.63	0.000
Suburban or moderate-sized city (population 50,000 to 250,000)	22.86	14.01		3.91	3.49		15.47	
Rural area or small city (population less than 50,000)	22.86	12.20		4.06	3.24		10.29	

Note: The sample excludes neurologists who have limited patient care responsibilities. Specifically, the sample excludes neurologists who provide direct patient care for less than 40 weeks per year and neurologists who do not have a clinical practice.

*Wilcoxon-Mann-Whitney tests were calculated for independent variables with two categories. Kruskal Wallis tests were calculated for independent variables with three or more categories.

**Chi-square tests or, as necessary due to small cell sizes, Fisher's exact tests were performed.

^†^MS subspecialists answered “Yes, and I chose a fellowship in MS” to question “Did you consider a fellowship in MS as subspecialty training?” and, on average, saw at least one MS patient per week. Respondents who did not complete an MS fellowship were categorized as MS subspecialists if indicated that they saw, on average, more than 12 MS patient seen per week.

^‡^The “other” category includes neurologists who responded “staff-model HMO,” “government hospital or clinic,” or “other public or private hospital or clinic setting” to question “Indicate in which practice arrangement you spend the majority of your clinical time.”

**Table 3:** Negative binomial regression predicting MS patients seen per week among MS subspecialists.

	Incidence rate ratio	95% confidence interval	*P* value
Year began medical practice (1972 to 1985 reference)			
1986 to 1993	0.72	0.51–1.01	0.060
1994 to 2001	0.78	0.56–1.09	0.147
2002 to 2011	0.74	0.46–1.19	0.212
Care involving a multidisciplinary approach (no reference)			
Yes	1.12	0.90–1.41	0.316
Personal connection to individuals with MS who are not your patients (no reference)			
Yes	1.07	0.76–1.50	0.714
Enjoy interacting with MS patients (no reference)			
Yes	1.63	1.31–2.03	0.000

**Table 4:** Negative binomial regression predicting MS patients seen per week among Non-MS subspecialists/general neurologists.

	Incidence rate ratio	95% confidence interval	*P* value
Subspecialty status (nonsubspecialist reference)			
Subspecialist	0.89	0.75–1.05	0.181
Practice arrangement (solo practice reference)			
Neurology group	1.18	0.96–1.46	0.118
Multispecialty group	1.09	0.82–1.45	0.556
University based group	0.59	0.41–0.85	0.005
Other^‡^	0.85	0.65–1.13	0.261
Unknown	0.76	0.57–1.01	0.055
Age (44 years or less reference)			
45 to 53 years	1.05	0.83–1.32	0.693
54 to 59 years	1.14	0.90–1.45	0.268
60 years or more	0.94	0.74–1.20	0.613
Sex (male reference)			
Female	0.81	0.66–0.99	0.039
Insufficient reimbursement for time involved (no reference)			
Yes	0.92	0.78–1.09	0.341
Lack of sufficient knowledge to feel comfortable caring for this patient population (no reference)			
Yes	0.51	0.29–0.92	0.025
Lack of sufficient knowledge regarding newer disease-modifying drugs (no reference)			
Yes	0.80	0.58–1.09	0.155
Seldom encounter MS patients (no reference)			
Yes	0.42	0.28–0.62	0.000
Ability to improve patient outcomes and quality of life (no reference)			
Yes	1.44	1.07–1.92	0.015
Dynamic area with evolving treatment options (no reference)			
Yes	1.27	1.04–1.54	0.017
Care involving a multidisciplinary approach (no reference)			
Yes	1.03	0.86–1.23	0.744
Research opportunities (no reference)			
Yes	1.18	0.95–1.47	0.125
Enjoy interacting with MS patients (no reference)			
Yes	1.41	1.18–1.67	0.000
Area in which you are practicing (within a major city reference)			
Suburban or moderate-sized city	1.18	0.98–1.41	0.076
Rural area or small city	1.30	1.02–1.65	0.031

^‡^The “other” category includes neurologists who responded “staff-model HMO,” “government hospital or clinic,” or “other public or private hospital or clinic setting” to question “Indicate in which practice arrangement you spend the majority of your clinical time.”
