# Supplementary material for: Factors Associated with Neurologists' Provision of MS Patient Care
Source: Mult Scler Int. 2014 Apr 24;2014:624790. doi: 10.1155/2014/624790 (PMC4020205; doi:10.1155/2014/624790)
Supplement: Supplementary file 1 — MS Physician Workforce Neurologist Survey The MS Physician Workforce Neurologist Survey was developed based on previous physician surveys, feedback from this study's Advisory Panel, and comments from members of the American Academy of Neurology (AAN) MS Section Executive Committee. This survey collected information from practicing neurologists on attitudes towards providing care for individuals with MS; attitudes towards neurologists who subspecialize in MS care; interactions with MS patients during residency training; current practice characteristics; and professional life satisfaction. [file 624790.f1.pdf]

Multiple Sclerosis Workforce Neurologist Survey

A. Care for MS Patients

1. **Which of the following factors play a role in limiting the number of MS patients you see in your practice?** (Mark all that apply)

- ☐ Care for MS patients takes too much time
- ☐ Insufficient reimbursement for time involved
- ☐ Lack of sufficient knowledge to feel comfortable caring for this patient population
- ☐ Lack of sufficient knowledge regarding newer disease-modifying drugs
- ☐ Lack of special personnel (nurses, social workers, etc.)
- ☐ Little can be done to improve MS patients' outcomes
- ☐ Seldom encounter MS patients
- ☐ MS patients are often difficult to treat
- ☐ MS patients often have multiple comorbidities
- ☐ Providing care to MS patients is not sustainable in my practice environment due to time or reimbursement constraints
- ☐ Other (please specify): ____

2. **Which of the following factors have a positive influence on your desire to provide care to MS patients in your practice?** (Mark all that apply)

- ☐ Ability to improve patient outcomes and quality of life
- ☐ Dynamic area with evolving treatment options
- ☐ Care involving a multidisciplinary approach
- ☐ Research opportunities
- ☐ Personal connection to individuals with MS who are not your patients
- ☐ Enjoy interacting with MS patients
- ☐ Community of dedicated professional colleagues with which to interact
- ☐ Other (please specify): ____

3. **Please rate your agreement or disagreement with the statements below.**

Compared with neurologists who don't specialize in the care of individuals with MS (including general neurologists and neurologists with other subspecialties), subspecialization in the care of individuals with MS provides neurologists with:

	Strongly disagree	Slightly disagree	Neither agree nor disagree	Slightly agree	Strongly agree
a. Increased academic employment opportunities	○	○	○	○	○
b. Increased private practice opportunities	○	○	○	○	○
c. Opportunities to do both patient care and research	○	○	○	○	○
d. Opportunities to provide direct patient care	○	○	○	○	○
e. The opportunity to teach/work with medical students, residents, and/or fellows	○	○	○	○	○
f. A patient population with unique medical and intellectual challenges	○	○	○	○	○
g. The opportunity to perform diagnostic tests and procedures	○	○	○	○	○
h. Opportunities to participate in drug studies	○	○	○	○	○
i. Opportunities to earn income comparable to general neurologists or those in other neurology subspecialties	○	○	○	○	○
j. The ability to improve outcomes for patients	○	○	○	○	○
k. High prestige compared to other medical specialties or subspecialties	○	○	○	○	○
l. The ability to balance personal/professional life (i.e., work/life balance)	○	○	○	○	○
m. A network of high quality peers	○	○	○	○	○

	Strongly disagree	Slightly disagree	Neither agree nor disagree	Slightly agree	Strongly agree
n. Enhanced job security	○	○	○	○	○
o. Sufficient numbers of patients to have a successful practice	○	○	○	○	○

4. **For this survey, an MS subspecialist is defined as a neurologist who focuses a substantial part of his or her clinical activities on care for individuals with MS. Using this definition, do you consider yourself an MS subspecialist?**
☐ Yes
☐ No

B. Residency and Subspecialty Training

5. **In what year did you begin medical practice after completing your medical training (i.e., after completing all specialty and subspecialty training)?**

_______ Year

6. **During your residency, did you:**

	Yes	No	Don't know/remember
a. Interact with any MS specialists	○	○	○
b. Participate in a MS clinic or with a MS care team	○	○	○
c. Participate in a research project related to MS	○	○	○
d. Participate in any neurology research	○	○	○

7. **Have you completed any neurology subspecialty training?**
☐ Yes, I completed a neurology subspecialty fellowship. Please list clinical focus of fellowship: _______________
☐ Yes, I participated in informal mentoring or training in a subspecialty area of neurology but did not complete a formal fellowship. Please list the primary clinical focus of this informal subspecialty training: _________________________
☐ No, I have not had subspecialty training

8. **When did you decide whether or not to pursue subspecialty training (which could include formal fellowships or more informal mentoring/training opportunities)? Indicate when you made your decision, regardless of whether or not you had subspecialty training.**
☐ Before medical school
☐ During medical school
☐ During residency
☐ After practicing general neurology
☐ After practicing in a different area of medicine

9. **How important were the following factors in deciding whether or not to pursue subspecialty training?**

	Not at all important	Slightly important	Somewhat important	Very important	Extremely important
a. Medical content of subspecialty	○	○	○	○	○
b. Availability of open spots in subspecialty training programs	○	○	○	○	○
c. Training program location	○	○	○	○	○
d. Potential for increased income	○	○	○	○	○
e. Amount of your education-related debt	○	○	○	○	○
f. Influence of mentor/teacher/role model	○	○	○	○	○
g. Personal/family member or friend's experiences with specific medical conditions	○	○	○	○	○
h. Loan repayment opportunity	○	○	○	○	○
i. Length of training program	○	○	○	○	○
j. Other (please specify):	○	○	○	○	○

	Not at all important	Slightly important	Somewhat important	Very important	Extremely important
______________________________					

10. **Did you consider a fellowship in MS as subspecialty training?**
- ☐ Yes, and I chose a fellowship in MS
- ☐ Yes, but I chose a fellowship in another area
- ☐ Yes, but I chose not to do a fellowship
- ☐ No

11. **Would you recommend MS subspecialization to medical students or physicians in training?**
- ☐ Yes
- ☐ No

C. Practice Characteristics

12. **Which best describes the area in which you are practicing?**
- ☐ Within a major city (population greater than 250,000)
- ☐ Suburban or moderate-sized city (population 50,000 to 250,000)
- ☐ Small city (population less than 50,000)
- ☐ Rural

13. **How many weeks per year do you provide direct patient care?** (Direct patient care is defined to include patient encounters, medical record review, consultation with clinical staff, discussion with family, telephone contact with patients, and resident supervision.)

_______ Weeks per year

14. **Which of the following best describes your patient care practice status?**
- ☐ I cannot accept any additional patients
- ☐ I can accept some additional patients
- ☐ I can accept many additional patients

15. **What is the current waiting time for a new patient visit in your practice?**
- ☐ 1-7 days
- ☐ 8-14 days
- ☐ 15-21 days
- ☐ 22-28 days
- ☐ 29-60 days
- ☐ More than 60 days

16. **How many total and new patients (including MS patients) do you see in an average week?**

	All patients	MS patients
a. Total number of patients seen in an average week		
b. Total number of new patients seen in an average week		

17. **For approximately how many MS patients are you the primary neurologist directing care?**

_______ Number of patients serving as primary MS care provider

18. **Do you provide consultation to other physicians regarding the care of MS patients?**
- ☐ Yes - Go to question 18a
- ☐ No - Skip to question 19

18a. **For approximately how many patients do you provide these consultations per month?**

_______ Patients per month

19. **How satisfied are you with the following areas of your professional life?**

	Not at all satisfied	Slightly satisfied	Somewhat satisfied	Very Satisfied	Extremely satisfied
a. Your career in medicine	○	○	○	○	○
b. Your medical specialty	○	○	○	○	○
c. Your medical subspecialty	○	○	○	○	○
d. Your current position	○	○	○	○	○
e. Relationships with colleagues	○	○	○	○	○
f. Relationships with patients	○	○	○	○	○
g. Personal time off (work/life balance)	○	○	○	○	○
h. Pay	○	○	○	○	○

20. **In the next 12 months, do you plan to retire or significantly reduce your patient care hours?**
- ☐ Yes, I plan to retire from patient care
- ☐ Yes, I plan to significantly reduce patient care hours
- ☐ No

21. **In the past year, have you or your institution/practice attempted to hire a neurologist to provide care primarily for MS patients?**
- ☐ Yes - Go to question 21a
- ☐ No - Skip to question 22

21a. **How long did it take to find an appropriate neurologist?**

_______ Months

22. **Please provide any additional comments that you would like to share on the topic of this survey.**

____

____

____
